# ‘A learning process that never ends’: How advantaged social justice activists negotiate privilege and activism within their identity

**DOI:** 10.1111/bjso.70077

**Published:** 2026-04-02

**Authors:** Frank Eckerle, Carmen S. Lienen, J. Christopher Cohrs

**Affiliations:** ^1^ Philipps‐Universität Marburg Marburg Germany; ^2^ University of Hagen Hagen Germany

**Keywords:** allyship, identity process theory, identity threat, privilege, social representations theory

## Abstract

Research shows that critically reflecting on ingroup privilege can motivate allyship. However, we lack a deeper understanding of how activists make sense of their privilege, how it contributes to their motivation to stay engaged, and how activism recursively affects the meaning‐making of social privilege. Building on social representations and identity process theory, we explored the social representation of privilege among allies and the identity processes involved in reconciling with ingroup privilege. We conducted 15 semi‐structured interviews with advantaged social justice activists (i.e., activists who are working in organizations to improve conditions for disadvantaged and oppressed groups). Applying thematic network analysis, we found convergent social representations of privilege but varying representations of its (systemic) roots, three types of identity threat elicited by privilege (*morality, positionality* and *social threat*) and four ways in which privilege relates to activism (*privilege enables action, privilege is a responsibility to act, quest for meaning* and *relativizing the role of privilege for activism*). A key insight concerns the prominent role of the coherence motive, which seems to help (re‐)conceptualizing privilege threat(s) in a way that motivates dismantling systems of inequality. We discuss the need for further theorizing on the bidirectional link between allyship and privilege reflection.

## INTRODUCTION

Social inequality and discrimination are defining features of our globalized society (Sidanius et al., 2001). This means that some people, by virtue of their intersecting identities or class background, receive a considerable number of unearned advantages, which can be compared to an invisible account of perks to spend (McIntosh, 1988, 2009, 2015). Social privilege can be defined as a set of special advantages that are granted by societal structures and are therefore easy to ignore or overlook for those who profit from them (Black & Stone, 2005). Social privilege is not static but constantly re‐made and maintained through everyday actions of *privileging* (e.g. treating people from privileged groups with more respect; see Minarik, 2017). The failure to recognize ingroup privilege is theorized to be rooted in a kind of motivated denial (Knowles et al., 2014): Hegemonic ideological beliefs in Western cultures tend to justify inequality through merit (e.g. Granaglia, 2019; Heuer et al., 2020), rendering ingroup privilege a threat to positive identity (Knowles et al., 2014). This explains why people often downplay (Täuber & Moughalian, 2022) or react defensively to confrontations with their social privileges (Eckerle et al., 2023; Knowles & Lowery, 2012; Shuman et al., 2024). Yet, some people who regularly experience privileging devote significant parts of their life to social justice activism and thus contribute to dismantling the very systems of inequality from which they profit (Pease, 2010).

Social psychological research has recently picked up interest in the link between social privilege and political solidarity or allyship. Allyship refers to advantaged group members actively dismantling systems of inequality and oppression (see Radke et al., 2020), which can be motivated by privilege awareness. For instance, the influential 3D model and its 4D extension (Knowles et al., 2014; Shuman et al., 2024) categorize the possible reactions to privilege as denying or defending privilege, distancing the self from privilege or from the privileged ingroup and dismantling systems of inequality (see Bergkamp et al., 2022, for a similar classification of reactions). Whether people then approach or avoid their social privilege is determined by two different kinds of threat that privilege reflection triggers: group‐image threat (i.e. through negative evaluation of the ingroup) and meritocratic threat (i.e. through loss of status justification). The 3D/4D model assumes that high levels of group‐image threat combined with low levels of meritocratic threat are most likely to motivate behaviour aimed at dismantling inequality (i.e. allyship). While Knowles et al. (2014) see allyship as an attempt to rectify negative group‐image, others argue that politicization in response to privilege can be understood as a process of disidentification with the ingroup and simultaneous identification with a politicized group (Bergkamp et al., 2022; Goodman, 2011; see also Subašić et al., 2008). Recently, it has been proposed that such outgroup‐ and ingroup‐focussed motivations can co‐exist (in conjunction with other motives), and that they predict different behaviours directed at the outgroup, spanning from (sometimes detrimental) helping to allyship (Radke et al., 2020).

In sum, previous research suggests that the meaning‐making of privilege affects the process of politicization and vice versa. However, to our knowledge, research has yet to investigate the role of privilege among people who are already engaged in social justice activism (i.e. allies). Given the surprising lack of qualitative inquiry into the experience of social privilege among social justice activists, we aim to acquire a deeper understanding of their social representations of social privilege (Moscovici, 1961) and the identity processes involved when integrating these representations into their activist identity (Breakwell, 1993; Jaspal & Breakwell, 2014). In other words, the present research situates the social representation of social privilege within the identity of social justice activists and investigates how the social representation of social privilege contributes to activists' continued motivation but also how privilege representation is affected by social justice activism.

### Social representations of privilege and identity motives

Social representations theory (Moscovici, 1961) posits that people construct shared meaning around social objects, which helps them communicate, coordinate and act in relation to that object. *Objects* can refer to different things, such as actual objects (e.g. electric cars), other social groups, societal structures or concepts (e.g. inequality or privilege). In other words, social representations depict what an object *means* to different (groups of) people.

To better understand what determines the content of a social representation and under which circumstances people are more likely to adopt certain social representations, scholars have integrated identity approaches into social representations research and argued for a bi‐directional relationship between the two (Breakwell, 2014). In one direction, social representations inform collective identities and group boundaries by fostering a shared reality and group consciousness among the members of a group. In the other direction, a person's group membership/identity influences what kind of social representations they are exposed to and whether they accept them (e.g. because they serve specific identity needs).

Social representations are shared within a broader group but this does not mean that they are identical for every group member. That is, identity processes at the intra‐ and interpersonal level may result in customisations of social representations (Breakwell, 2014). Investigating this interface between *social* representations (i.e. aggregated meaning‐making of an object) and *individual* representations (i.e. beliefs about an object that become part of one's identity) is a core feature of identity process theory (IPT; Jaspal & Breakwell, 2014).

IPT builds on social representations theory and social identity theory (Tajfel & Turner, 1986). More than other identity theories, IPT focusses on psychological change by assuming two universal identity processes that people go through when they encounter new information that is relevant to them. The process of assimilation‐accommodation describes the reception of new information (e.g. learning about privilege) as well as the adjustment to this new information (e.g. changing the beliefs about oneself *or* rejecting the information as irrelevant). The second process, evaluation, describes the effort of making sense of and assigning value to the new identity content (e.g. how to be a good, privileged social justice activist). Identity processes thus take place in a world of social representations, that is, different understandings about societal structures (e.g. inequality), social groups and so on. The individual engages with these representations and, according to IPT, leans towards adopting and integrating those that result in the least identity threat (see Jaspal, 2014).

The two identity processes are guided by four (plus three) identity motives, namely *continuity* across time and situation, *distinctiveness* from others, *self‐efficacy* (feeling confident and in control of one's life) and *self‐esteem* (feelings of personal and social worth; Jaspal, 2014). Research has also suggested additional identity motives, such as *belonging* to other people, living a *meaning*ful life (Vignoles et al., 2002), and *coherence*, the motive of bringing different identities and life aspects into accordance (Jaspal & Cinnirella, 2010). In sum, each of these seven identity motives plays a part in the constant (re‐)construction and maintenance of identity (Breakwell, 2014). By tracing how these identity motives become threatened, we aim to uncover the process of assimilation–accommodation of social privilege with regard to the identity of advantaged social justice activists.

### Making sense of privilege *through* allyship?

There are many paths to allyship and negotiating social privilege is a key one (Radke et al., 2020). The Model of Integrating Awareness of a Privileged Social Identity (Bergkamp et al., 2022), for instance, specifies that the critical exposure to privilege and systems of inequality elicits identity threat which in turn motivates identity protection strategies. Furthermore, intrapersonal, interpersonal and cognitive resources can help bypass identity protection motives and help reconcile the self with one's social privilege. The model implies that the path to allyship is non‐linear and recursive: People may experience new identity threats even after having reconciled with privilege before (e.g. when a male White ally is confronted with reproducing patriarchy in the movement). However, previous research has not yet investigated how the meaning‐making of privilege relates to the lived experience of allyship and which identity threats and processes are relevant in reconciling social privilege with activist identity. In other words, while previous research has focussed on the motivating factors behind *becoming* an ally, we focus on the social identity processes at work while *being* an ally. We argue that social representations and identity process theory are well situated to trace the ongoing negotiation between one's understanding of social privilege and one's motivation to act in solidarity to dismantle the systems from which they stem. In sum, we adopt a process ontology to explore the continuously evolving relationship between meaning‐making regarding social privilege and activist identity.

## METHOD

### Participants

We recruited participants by sending invitations to roughly 100 different activist organizations and their local groups in Germany between January 2020 and September 2021. The invitation introduced our study as an investigation of the motives behind political solidarity and did not mention the politically charged term ‘privilege’ (see Quarles & Bozarth, 2022). One participant was further recruited via personal contacts. As an incentive for participation in the study, we offered a short summary of our findings as inspiration for further individual or group‐based self‐reflection.

People were eligible to participate if they were at least 18 years old, spoke German fluently and were currently active in a political organization for social justice. To ensure that they were in fact *advantaged* activists, we specified in the invitation that the organization they were working for should be politically engaged in supporting disadvantaged groups they do not personally belong to.

Some difficulties during data collection should be pointed out: First, the recruitment took place during the height of the COVID‐19 pandemic, which made face‐to‐face interviews impossible. Second, social justice activists often live on a full schedule, which is reflected in the high risk for burn‐out in this group (Chen & Gorski, 2015). As such, the response rate to our email invitations was low. In total, we recruited 16 participants. One interview was discontinued and its content was not transcribed because the participant did not fit the advantaged activist criteria (she had a physical disability and was working for the rights of the disabled). The final sample thus consisted of 15 interviews (10 women, 5 men, age range = 23–76, median = 29 years). All participants identified themselves as White. 14 interviewees worked in German and one in a South African organization(s). Consult Table 1 for further information.

**TABLE 1:** Participant information.

Number	Alias	Age	Gender	Organization	Role/main activity
1	Christian	28	M	Seebrücke	Political campaigns
2	Sebastian	29	M	Seawatch	Crew on ship
3	Luisa	29	W	Youth Camps and Anti‐Racism Trainings	Anonymous
4	Ute	42	W	Seebrücke	Organizing team
5	Carla	24	W	Seebrücke	Diversity team
6	Lina	27	W	Seebrücke	Coordination team
7	Rolf	76	M	Amnesty International	Various
8	Margarete	33	W	Anonymous	Anonymous
9	Renate	51	W	Amnesty	Coordination
10	Alex	69	M	Amnesty	Pro‐refugee work
11	Oliver	31	M	Seebrücke	Organizing team
12	Sina	28	W	Anti‐discrimination office	Counselling
13	Carola	27	W	Seebrücke	Lobbying
14	Charlie	23	W	Zugvögel	Organizing team
15	Marco	36	M	Lesbian and Gay Association Germany	Anonymous

*Note*: All names are pseudonyms. Participants were asked openly to self‐identify their gender; M, man; W, woman. More information about the bigger organizations are presented in the Supplementary Materials S1.

The study was reviewed and approved by the Research Ethics Committee of the Philipps‐Universität Marburg. Written informed consent was obtained from all participants prior to the interviews.

### Data collection

The semi‐structured interviews consisted of three thematic parts, which were subdivided into follow‐up questions to gather deeper insights. The first part featured questions on the personal history of participants' political activism as well as their former and current motivation behind it. The second part consisted of inquiries regarding the subjective meaning of privilege and the (personal) feelings and thoughts associated with being privileged. In the third part, we tied the previous themes together and asked directly in what way(s) their privilege does or does not play a role in their political activism. Due to the COVID‐19 pandemic, most interviews were held remotely via telephone or voice over IP. The first two authors and one Master's student conducted the interviews.^1^

Interviews and their audio‐recordings lasted between 30 and 90 min. Audio files were transcribed verbatim based on semantic content‐related transcription rules (Dresing & Pehl, 2018). We used strict anonymization rules to delete personal information and identifiers from the transcripts before coding.

The interview guide can be reviewed in the Supplementary Materials S1. The main questions presented in the guide were always asked. We monitored whether participants responded to additional questions unprompted and asked these questions only if deemed necessary. Note that questions about privilege appeared *after* questions about motivations for political activism in the interview guide. This was done to allow for the possibility that participants mention their privilege as a factor within their activism unprompted, thus providing more insight into the explicit role privilege plays within their activist identity and avoiding suggestive bias on our part.

Seven of the 15 activists mentioned their privilege unprompted and related it to their activism (Christian, Sebastian, Luisa, Margarete, Sina, Charlie and Marco). Three mentioned related issues of positionality or responsibility based on their social status, without directly using the word privilege (Carola, Renate, Ute). In the section where we asked about privileges, only one activist (Rolf) denied their existence.

### Data analysis

**FIGURE 1:**
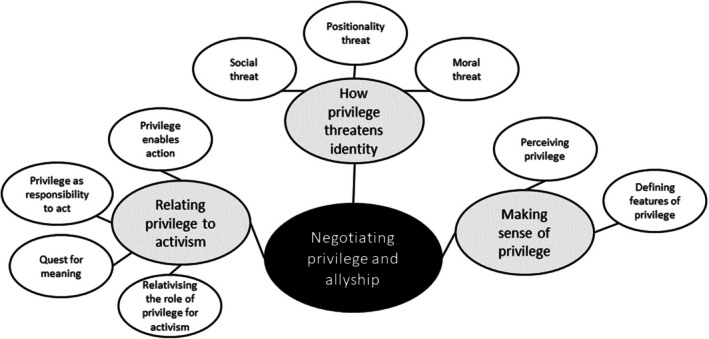
Thematic network: Negotiating privilege and allyship.

For the analysis of the interviews, we applied Thematic Network Analysis (Attride‐Stirling, 2001) and used MAXQDA 2024 software (VERBI Software, 2025). Building on approaches such as grounded theory (Corbin & Strauss, 1990) and argumentation theory (Toulmin, 1958), Thematic Network Analysis aims to ‘explore the understanding of an issue or the signification of an idea’ (Attride‐Stirling, 2001, p. 387). Like many other qualitative analysis approaches, it constitutes a tool to identify and organize relevant arguments or issues within qualitative data. We chose Thematic Network Analysis because it provides a structured approach to data analysis that allowed us to focus on shared understandings across interviewees as well as ‘differences or contradictions’ within the data (Attride‐Stirling, 2001, p. 365), which we found particularly useful for our research question on how advantaged social justice activists reflect on their privilege. The first two authors first read and re‐read the interview transcripts multiple times to familiarize themselves with the data and to discuss potential prevalent themes. The first author then coded the interviews following the suggested procedure in Attride‐Stirling (2001), which entailed establishing Basic Themes that express ideas or arguments close to the text, categorizing those into Organizing Themes that constitute a more abstract interpretation of the ideas underlying the Basic Themes, and grouping these Organizing Themes into superordinate Global Themes. Themes are then presented in the form of a network that reflects the interconnectivity between them (see Figure 1). Finally, the second author provided *credibility checks* (i.e. verifying themes and interpretations; Elliott et al., 1999), with the third author providing additional thoughts and comments. Any disagreements were resolved in discussions until a consensus was reached.

The analysis was informed by the data as well as through the theoretical lens of IPT. Analytic procedures that allow flexibility concerning the implementation of theory into the analytic process, such as Thematic Network Theory, have been discussed as particularly useful for identity process theory research (Coyle & Murtagh, 2014). Specifically, we coded sections that we interpreted as reflecting identity motives underlying participants' responses towards their own privilege or their activism in addition to other relevant themes. We then grouped themes according to the insights they provide on the meaning‐making of privilege, activist identity, and the link between them.

## RESULTS

We identified three global themes which together provide information on the social representation of privilege among advantaged social justice activists (*Making sense of privilege*), associated identity threats (*How privilege threatens identity*) and its relation to their social justice activism (*Relating privilege to activism*). These themes in turn consisted of several Organizing and Basic themes. Conceptually, the thematic network captures the process of meaning‐making of privilege, the relevant identity threats associated with privilege, and the way privilege is integrated into the activist identity. The suggested structure of global and organizing themes is represented in Figure 1.

### Making sense of privilege

The global theme *Making sense of privilege* captures social representations of social privilege. Here, activists focussed on trying to make sense of the concept of privilege, how it is defined and how and under which circumstances the concept appears relevant.

#### Defining features of privilege

##### Privileges are unearned advantages

Participants generally shared an understanding of social privilege as a collection of unearned or unjustified advantages, for example, ‘things that I have done nothing for that make my life easier’ (Sina, 28). These advantages were generally understood to be based on their social identity, for example, ‘based on a certain background or on certain properties, which are ascribed from the outside’ (Christian, 28). However, participants did not differentiate much between different advantaged social identities (White, middle‐class, heterosexual, etc.). In contrast, social privilege was predominantly seen as a combination of the socially advantaged groups one belongs to, reflecting an intersectional perspective. Still, participants often explained how specific instances of social privilege manifest, such as when Carla (25) shared that she understands her White privilege as a freedom from disadvantage, ‘That I, yes, am not constantly checked by the police. That I'm not denied that I'm German or that I grew up in this country or that I belong here because of my appearance’. (Carla, 25).

While participants largely agreed on what constitutes social privilege, there was considerable diversity with regards to the participants' understanding of its origin and how they personally relate to its maintenance. Central to these accounts was a tension between conceptualizing social privilege as rooted in contingency and understanding it as structurally produced. For some, privileges were mainly reasoned to be rooted in chance, for example as, ‘luck in the birth lottery’ (Renate, 51). While this representation acknowledges that privilege is unearned, it also emphasizes an individualized account of privilege that is disconnected from the structural processes that contribute to the ongoing process of privileging (Minarik, 2017). An individualized account of privilege also aligns with a meritocratic worldview according to which people are judged by what they make of the cards they have been dealt. Other activists emphasized the structural nature of privileging, for instance, ‘for me these are simply societal structures, such as that people with a certain background or certain characteristics, which are also often ascribed from the outside, which are *made to be meaningful* […], such as things like skin colour, religion, sexual orientation’ (Christian, 28), or put more simply: ‘not to be implicated by structural disadvantaging’ (Sebastian, 29). These more structural representations of privilege included an understanding of privilege as socially constructed.

Participants placed different emphasis regarding their own role in upholding this social construction, adopting either a unidirectional representation by emphasizing the role of unfair systemic arrangements or a more complex representation by emphasizing the constant reproduction of hegemony and power. The following quote by Marco (36) seems to reflect a view according to which social status and privilege mainly flow from society to the individual or group: ‘So basically, it does have something to do with what my social status is. Where do I come from? Which opportunities do I have? I think that can definitely make a difference’ (Marco, 36). Others more strongly emphasized that those with social privilege also maintain (willingly or not) the normativity of inequality, which Luisa (29) referred to as ‘interpretive authority’ and which Sina (28) put the following way:

Being a man is the norm and being a woman is the deviation […] And as soon as a person's characteristic is seen as a deviation, it becomes more difficult, because barriers are put in the way. And that has a lot to do with who has the resources and power to enforce things and construct themselves as the norm. (Sina, 28).

While participants were generally aware of their social privilege, they engaged with the concept of privilege in ways that foregrounded different aspects while leaving others less visible. These variations point to ongoing tensions in how privilege is made sense of between chance and structure and between systemic constraint and individual agency.

##### Deviate representations of privilege and uncertainty

While the previous section illustrated shared reference points as well as internal tensions in how privilege was understood, a small number of accounts departed more fundamentally from this frame. Rather than differing in emphasis or complexity, these accounts were characterized by uncertainty or outright rejection of the concept itself.

In one instance, Rolf (76) argued that privileges cannot exist within democracies because privilege was a specific element of feudalism and the ‘corporate state’, which had been demolished in modern democracies. Additionally, Ute (42) and Renate (51) admitted that it was complicated to pin down what social privilege is and what it is not

I don't know enough about privileges, it's really a question of what you mean by privileges. […] Without giving it any thought, I would like to see them abolished. But I haven't really thought about it enough, not at all, hardly at all. So, I can't say. (Ute, 42)

It is noticeable that Ute, who worked in the field of social justice for many years, has not engaged with the concept of privilege. This could potentially be explained as a generational effect, according to which older generations are less prone to reflect on their social privilege than younger generations (e.g. Egan Brad et al., 2019). While to several of our participants there seemed to be a shared understanding that whatever privilege is, it is something bad that should be dismantled, some interviewees found the concept overwhelming. Ute, who admitted that she had not yet dealt much with privileges, at a later point in the interview voiced an interest in learning more about this concept. In her interview, it became apparent that she was uncomfortable with her lack of knowledge, possibly because she felt she needed to be better informed in her role as an activist. Rolf, in contrast, deemed privilege an irrelevant concept. These deviate representations and uncertainties suggest that a clear representation of social privilege is not a necessary factor for motivating social justice activism. However, lack of privilege awareness can threaten the collective effort via the (unconscious) reproduction of power hierarchies within the movement (see also Radke et al., 2020).

#### Perceiving privilege

##### Privileges are hidden

Interviewees generally shared the belief that people are ‘not really aware about how privileged they are’ (Carola, 27), and that privileges are often perceived as ‘a given’ (Marco, 36), because they feel like ‘normality’ (Christian, 28). Seven activists in our sample also pointed out that the belief in meritocracy, or its German term *Leistungsgesellschaft*,^2^ curtails privilege perceptions: ‘Well, the majority of society assumes that positions have been earned and that we are a meritocracy’ (Sebastian, 29). In a similar vein, Carola (27) described that she was more likely to see inequality if she walked through the city wearing her metaphorical ‘privilege glasses’, meaning when she made a conscious effort to notice them. These accounts align with the definition of privilege as usually outside of the awareness of its benefactors (Black & Stone, 2005) and as obscured by meritocratic ideology (Knowles et al., 2014), which is the belief in a ‘social system in which advancement in society is based on an individual's capabilities and merits’ (Kim & Choi, 2017, p. 112).

In some cases, the myth of meritocracy (e.g. Trevisan et al., 2022) also seemed to obfuscate privilege to the participants. For instance, Renate (51) and Marco (36) argued that privilege was attainable through merit, ‘You can earn them […]. I've been in the job I do for quite a long time. I helped build up the unit. And I have certain freedoms that others don't have’ (Renate, 51). In this case, privilege was understood as an individualized characteristic and the result of hard work, implying an understanding of privilege as rooted in meritocracy. In fact, the idea of ‘earning’ privilege reflects a *lack* of awareness about the group‐based and systemic nature of social privilege. Rooting privilege in meritocracy seems especially relevant within the German representation of meritocracy given that previous qualitative research has demonstrated it foregrounds the belief that social inequality is justified by superior work ethics (Heuer et al., 2020). Therefore, the idea of *earning privilege* may help reframe social privilege as fair and reduce identity threat (see Phillips & Lowery, 2020).

##### Witnessing inequality/discrimination

Another important theme concerned the question *under which conditions* privilege becomes perceptible to the privileged. There were multiple instances when interviewees recounted experiences in which confrontations with inequality highlighted their own privilege, such as when Margarete (33) realized ‘that people have to go through certain experiences of racism and I didn't realise that at all’ after reading a seminal book on racial inequality in Germany or when Carla (24) recounted a university seminar where the term privilege was first brought to her attention. Charlie (23) argued that contact with disadvantaged perspectives helps build privilege awareness:

And only when someone points it out and somehow gives you the impetus to think about it, perhaps only then does the idea occur to you that “it's not so obvious after all”. But I think if that doesn't happen, it's very easy to overlook. (Charlie, 23)

Sina (28) made use of an analogy by transferring her own experiences with sexism to make sense of the experience of racism despite her White identity, stating ‘that at least I have a reference point to say, ‘Okay, it's not completely the same, but I can relate to what you, what people who experience racism, for example, are facing’. In sum, participants highlighted that being able to perceive and to deal with social privileges requires critical education or critical experiences, either through contrasting one's own privileged position vis‐à‐vis a disadvantaged person or group or through interaction with other privileged group members.

### How privilege threatens identity

All interviewees reported in some form or another that privileges can be threatening to their identity. We categorized these threats into themes surrounding moral, positionality and social threat. Additionally, we relate the relevant identity motives to each threat to highlight the process by which each of these issues *becomes* threatening to the self.

#### Privilege as a moral threat

That privilege feels morally wrong and shines a bad spotlight on oneself was a particularly prevalent theme. Interviewees mentioned various reasons for this negative association. For instance, Christian (28) shared that there are certain experiences in which privileges become apparent, and which weigh especially heavy on his conscience:

I think it doesn't feel particularly good when you are made aware that you have these privileges and, fortunately, don't have to go through such experiences, but because other people do go through them and you don't have to compete with other people for a job, for example, you simply benefit from the system and are part of the system […] (Christian, 28)

This quote reveals a disparity between the 3D model's assumption that meritocratic threat (i.e. threat that one's status is partly undeserved) would be negatively related to the intention to dismantle inequalities (Knowles et al., 2014). Instead, Christian seemed to appraise the fact that his status was partly undeserved as a moral issue. The moral ramifications of privilege awareness are illustrated in the following account, where Charlie (23) remembered the shock that she experienced when she realized the advantages conferred to her through inequality and her implication in discrimination:

And on the other hand, I think […] reflecting on my own privileges is initially a bit of a startling moment, because I haven't seen it for so long, and I've also discriminated against other people in some ways. And that there's this moment of shock. (Charlie, 23)

There is some contention between Christian's and Charlie's accounts: While Christian mainly highlighted the identity threat inherent to his realization of profiting from an unjust system, Charlie more closely related identity threat to the realization of her own implication in and contribution to this system. In addition, participants, including Charlie, often referred to moments of shock or paralysis when confronted with their social privilege in places where they had not previously thought it was relevant. This resonates with the idea that some scaffolding (in the form of knowledge, social support, etc.) is needed to move from experiences of exposure with inequality and ingroup privilege towards privilege acceptance and allyship (Bergkamp et al., 2022).

Negative emotions, such as guilt and shame, play a prominent role in the context of identity threat generally (Gausel & Leach, 2011) and also identity threat through confrontations with social privilege specifically (e.g. Eckerle et al., 2023; Iyer et al., 2003). In line with this, participants shared various examples of guilt and shame while reflecting on ingroup privilege. For example, Luisa (29) associated privilege reflection with a ‘guilt component’ and Margarete (33) stated that reflecting on her own social privilege made her feel ‘dismayed and speechless’. In fact, the interview with Margarete had to be paused for a few minutes because the discussion around social privilege and systemic inequality became emotionally stressful for her. Sina (28) also highlighted that it takes effort and energy to manage feelings of shame when reflecting upon social privilege: ‘Well then, I have to keep getting out of this emotion [shame] so that I can have the energy to do something again’. The importance of overcoming negative emotions about the self is also reflected in Steele's (1990) seminal work on *White Guilt*, which concludes that ‘[s]elfish white guilt is really self‐importance’ (p. 506).

In line with this conjecture, negative emotions attached to social privilege sometimes seemed to motivate withdrawal from the topic. Oliver's (31) following candid account demonstrates the motivated evasion of the topic of privilege, both because it threatens identity (i.e. is connected to negative emotions) and because it has not been fully integrated into his activist identity (i.e. because he believes it distracts from achieving change).

I don't think it's anything I like to pre‐occupy myself with, privileges. […] And I don't really believe in it either, because it doesn't achieve anything and it's emotionally uncomfortable. But perhaps there is also this latent fear that I have, that someone will somehow see me as guilty because I am somehow privileged. And I think this fear is also there because I think, “Okay, as someone who is privileged you get confused with someone who oppresses or exploits or dominates in some way” (Oliver, 31)

To Oliver social privilege seems to represents a moral threat, because to him privilege frames him as someone who *actively* oppresses others. In this sense, the quote indicates that his allyship motivation may be rooted in a need for personal moral acceptance (Radke et al., 2020). He also implies that the discourse around social privilege gatekeeps ‘good activism’ by associating privilege with oppression. This framing threatens to individualize oppression as a matter of voluntary action rather than as a structural condition that implicates all advantaged group members, regardless of their intent. The individualized understanding of social privilege is morally neutral or even irrelevant to social justice activism, allowing Oliver to distance himself from responsibility and rejecting the concept of social privilege as unhelpful. Such framings may be particularly consequential in collective action contexts, as they threaten to reproduce power hierarchies by shifting the focus from structural implication to personal moral innocence (Droogendyk et al., 2016).

Within an IPT framework, the present theme demonstrates that privilege can violate the self‐esteem motive (due to its negative connotation for how one sees oneself) and the coherence motive (due to the misfit between moral ideals and one's advantaged position or behaviour). It seems that the nature of threat is determined to some degree by the specific allyship motive, which can be rooted in ingroup, outgroup, personal or moral concerns (Radke et al., 2020). Even though one of the advantages allies enjoy is the ability to withdraw from reflections on social privilege (such as demonstrated by Oliver), our interviews showcase that activists can also choose the opposite strategy: acknowledging the immorality of their status and/or their implication in maintaining an oppressive system. From an IPT perspective, this strategy reflects an assimilation–accommodation process by which the acceptance of the immorality of social privilege scaffolds its integration into identity.

#### Privilege as a positionality threat

There was a recurring sense across the interviews that being privileged determines how one relates to the world and that social privilege would therefore define a person's experiences and, ultimately, their identity. We term this ‘positionality threat’ as it relates to a sense of inevitability of positionality in the context of systems of inequality (e.g. privileged people do not ‘choose’ their privileged identities). Positionality threat surfaced through the realization that privilege has implication for one's position in the wider system of inequality. Luisa (29) provided an example for this, which highlights the inevitability of immorality and reflects a critical and reflective stance: ‘It is said that in a system of discrimination and injustice, not only those whose humanity has been denied are unfree. But also, those who have actively taken humanity away from others’ (Luisa, 29). The quote reflects a representation of systems of inequality not as privileging some and oppressing others but ultimately harming everyone. This reframing situates the advantaged and the disadvantaged within the same boat of suffering from inequality—a perspective that may help forging a shared identity around a common enemy (e.g. elites fostering inequality) but may also obscure the material consequences of inequality (World Inequality Lab, 2026).

Alex (69) provided a different nuance of positionality threat, lamenting the impression that his status would hinder him from effective solidarity because he might be taken less seriously by disadvantaged groups (refugee families in his case). He further seeks to hide his privileged position from them more out of a feeling of guilt:

I have a lot of contact with refugee families here. When I see how they live, under what conditions, and how I live. Then I feel a little guilty towards them. That's why I avoid inviting them to my home. […] If they see how I live, under what circumstances, then I could imagine that in some cases they might not take my advice quite so seriously. […] They might say, “It's easy for you to say, you live like that. Live like me, then you wouldn't do that either.” (Alex, 69)

The quote reflects a reproduction of power hierarchies in which Alex gets to see the living conditions of refugee families while exerting his power to avoid them seeing his privileged living situation and therefore avoid feeling guilty. Contrary to Alex’ worries that the salience of status differential would negatively influence his support of refugees, research showed that when status inequality is emphasized within situations of cross‐group contact, this will not only decrease prejudice of the advantaged group, it may also increase collective action intentions among the disadvantaged group (Becker et al., 2013; Droogendyk et al., 2016).

Interviewees also shared that in some ways their privilege can limit the ability for personal growth due to a lack of critical experiences, for instance ‘Perhaps these are experiences that one sometimes lacks, experiences that may also make one stronger personally’. (Sebastian, 29). Here, Sebastian seems to consider a ‘what doesn't kill you makes you stronger’ perspective according to which structural disadvantage may be a source of individual development. However, this perspective can have problematic implications as it is prone to romanticize disadvantage.

#### Privilege as a social threat

We also identified instances of social threat, or threats to the belonging motive (i.e. the need for feeling socially connected to others). Social threat was especially relevant when discussing social privilege with other advantaged group members who did not share their critical perspective. For instance, Margarete (33) recounted a particularly negative conflict with peers where ‘the discussion went so badly that I cried at the end because there was totally this attempt, at least I had the feeling, that my friends were trying to ignore or minimize the issue’. Furthermore, Luisa (29) told us about the difficulty of trying to nudge others towards more critical perspectives and how it endangers harmony, for example, at work: ‘But in some areas where you know that people don't have a basic understanding, you can only nudge them gently, because otherwise it often ends up in an argument’. Addressing privilege can therefore threaten previously established positive relations by driving a wedge between those conscious of how systems of privileging shape their everyday life and those who do not. This conflict between advantaged group members on different levels of privilege awareness may be an underemphasized layer of contention potentially impacting advantaged group members effectiveness when working in solidarity with the disadvantaged. Yet, previous research indicates that privilege confrontations among advantaged members are an important vehicle for change. For instance, Eckerle et al. (2023) found that *cis*‐men reacted more positively to privilege confrontations by ingroup members (other *cis*‐men) than by disadvantaged outgroup members (e.g. women), which emphasizes the importance for the advantaged to confront privilege among their peers. Additionally, this perceived threat to belonging is experienced within a system that, by definition, exempts the privileged from the constant need to *prove* their belongingness to society.

For some, the belonging motive was further threatened by the disadvantaged groups' ostensibly stronger ability to achieve group consciousness and to build community, which led to feelings of exclusion:

But we are not alone and can fight back together or discuss it together, and a bit of an identity is getting created through this. Which I believe is often not so present in people who are privileged. […] A feeling of solidarity. Which comes a little bit from being in a position of discrimination and oppression. (Carla, 24)

Explicit in this theme is the idea that oppression is perceived as a source of identification which the privileged lack and, to some degree, miss. This perspective seems to stem from experiencing limited access to spaces of disadvantaged groups that allies identify with, especially given the contentious nature of their relationship with less privilege aware advantaged group members discussed above. However, social privilege is partly defined through preferential treatment between privileged group members (McIntosh, 1988). Therefore, the experience of alienation is more likely to stem from the fact that being shut out from social groups based on one's social identity is a relatively uncommon experience for advantaged group members. An additional explanation for the experience of social threat vis‐à‐vis the disadvantaged group(s) is rooted in neoliberal hegemony, which affects both advantaged and disadvantaged group's capacity of community building in different ways and to different degrees (see Adams et al., 2019).

### Relating privilege to activism

In association with the threats identified above, we found an array of links between the (threatening) meaning‐making of privilege and its consequences for social justice activism. In terms of IPT, these strategies can be understood as pathways that integrate privilege into the activist identity (privilege enables action, privilege as a moral responsibility to act or a quest for meaning) or to relativize its relevance for identity, although both strategies did sometimes exist within the same persons.

#### Privilege enables action

In line with previous research on the role of empowerment in activism (Drury & Reicher, 2005), privilege was sometimes reconceptualized as a source of agency and efficacy. For example, Lina (27) highlighted: ‘I think it builds on that. Activism builds on privilege. […] In the sense that if you are privileged, it is easier to get involved’.

This empowerment was connected to the activist identity in different ways. Some participants attributed the sheer capability to be politically active to their privilege, such as Marco (36), who reflected on his class privileges by imagining that after a long day of manual labour, ‘I might have less of an opportunity to be politically active, even though I might be a political person’. Furthermore, Oliver (31), who was rather critical of the use of privilege reflection for activism, conceded, ‘I find this question, ‘How can you use privilege to somehow help less privileged people?’ I actually find that kind of exciting’. These quotes demonstrate that the realization that social privilege provides the appropriate resources to become politically active and standing to be heard is an integral link between privilege and the activist identity.

Importantly, however, Sina (28) emphasized that the *ability* to intervene does not always mean that one *should* intervene. In line with discussions on the perils of advantaged allies in social movements (e.g. Droogendyk et al., 2016), she mentioned that it was important to attune her involvement to the desires and needs of those they are supposed to support. This view also seems to reflect the important differentiation between motives of self‐enhancement (i.e. self‐esteem/personal status) and self‐transcendence (i.e. universalism), which have quite different implications for how others react to allies (Phillips et al., 2024).

To see what my attitude is, but also what influence I have. And how can I use that to change society? Always, of course, in dialogue with the people who are affected. Because I can only see things through my White or other privileged lens. And that can't be in the interests of the people I actually want to stand up for. (Sina, 28)

In sum, privilege can be perceived as a form of empowerment that enables activism and renders it less threatening. This realization may then lead to the ‘empowering relief’ (Bergkamp et al., 2022, p. 13) that the unearned social status can be used for good, for instance, by engaging in allyship.

#### Privilege is a responsibility to act

Despite some conceding that their activism is unlikely to significantly change the status quo, because it ‘has been consolidated for a long time’ (Charlie, 23), interviewees often mentioned that having social privileges comes with the moral responsibility to act for social change or to at least do *something*. For example, Sebastian (29) stated, ‘I do think that, as a privileged person, you have a responsibility to work beyond the privileged group to ensure that others are able to do well. And that they can also have a good life’. It seems, then, that from the realization that privilege enables action (see above) flows the realization that privilege demands action.

##### From reflection to action

The importance of doing (vs talking) was illustrated by Margarete (33), who recounted that the urge to become active grew as she was researching antiracism during university:

I think it was definitely a good step to do something active, because I've distanced myself a bit from always writing about it and thereby also constraining identities and so on. (Margarete, 33)

This quote demonstrates awareness of the ongoing process of privileging (Minarik, 2017): that talking about privilege only within ones privileged ingroup can reproduce inequality by reducing the oppressed and disadvantaged to their role of victims instead of amplifying their voices. As Luisa (29) exemplifies, this awareness has ramifications for one's moral compass:

As someone who is as well off as I am and who is so well provided for, I actually think that you have a duty, a social duty, a moral duty, not to invest your own labour and time solely for a salary. Because it's not just about money. And that has always been important to me. […]. And I always think it's important to try and think a little bit about, “Where can I, how do I want to fulfil my responsibility?” (Luisa, 29)

Integrating social privilege into identity can therefore significantly raise moral stakes, because it renders working towards social justice a moral obligation or ‘duty’, which should not be dependent on outside praise or concerns for efficacy. However, the moral stakes can also become so high, that *doing the right thing* seems almost impossible. This is exemplified by the following quote:

And, yes, actually the obligations that come with privileges are at the forefront. And yes, we try, mostly without much success, to involve those who are less privileged. […] Not always, but mostly, there are White people in the crew and black people who are being helped in the situation, and that just reinforces the image of White saviours and black victims. And we don't want to reproduce that. (Sebastian, 29)

Here, Sebastian raises an important dialectic, highlighting that the responsibility to act includes concerns about the responsibility to act in a way that does not reproduce oppressive hierarchies and narratives (i.e. White saviourism). In fact, one considerable outcome of this thought process is that not acting may sometimes be preferential to acting. While this would certainly be detrimental in the context of Sebastian's engagement in sea rescue, research has shown in other contexts that the presence of allies within political movements has surprisingly little effect on their political success (Hartwich et al., 2023). Therefore, effective political solidarity entails knowing when to take a backseat from protesting and finding other ways to support, such as by taking supportive roles and by providing care work that enables others to concentrate on protesting or recovering from protesting.

Taken together, the present theme links privilege reflection to action via feelings of (moral) responsibility. This reflection also seems to include worries about how advantaged activism may be detrimental to the cause of those it seeks to support (e.g. by co‐opting voices or reproducing White saviour narratives and imagery). The question of whether or not to act seemed to be rather independent of efficacy considerations as it was often considered a responsibility to contribute to even small‐scale change.

##### Navigating the guilt—responsibility nexus

Consistent with the theme that privilege threatens identity, guilt reappeared in this theme as a contested link between privilege and responsibility. Sina (28), for example, strongly advocated leaving guilt out of the equation in favour of taking a stance of responsibility:

I find it really exciting to deal with this emotion a lot and say, “No, we have to make sure we take responsibility, and we can't do that if we feel guilty. It blocks us. So how do we get out of it”? (Sina, 28)

Sina seems to argue that to move from threatening guilt to allyship requires reappraisal of privilege not as guilt but as a responsibility; an idea that resonates with Bergkamp et al.'s (2022) allyship model, which differentiates between the stage of identity protection (e.g. motivated by guilt) and the stage of reconciliation (e.g. motivated by responsibility); and pedagogical approaches of *shared responsibility* (Zembylas, 2019) and *anti‐complicity* (Zembylas, 2020). Similarly, Carola (27) pointed out that responsibility of the privileged transcends individual guilt, ‘Because I'm not responsible for the fact that I have this privilege, nor that it's unfair. I can only try to change this injustice’.

Others went even further and rooted responsibility in a symbiotic and reciprocal relationship between the self, others and society, ‘I am convinced that when I do something for others, I am also doing something for myself. And at the same time, I am doing something for society’ (Marco, 36). In this account, Marco seems to navigate the guilt‐responsibility nexus by internalizing a group‐based moral motive of social justice based on the value of communal solidarity (see Janoff‐Bulman & Carnes, 2013). Together, these three accounts suggest that the meaning‐making of privilege as a social responsibility can provide clarity, and in some cases help move beyond perceiving individual fault, guilt and other identity threats.

#### Quest for meaning

Activists discussed their activism as something that can provide meaning to their life. Sebastian (29) summarized vividly the sentiment that leveraging privilege for allyship helped give his advantages meaning:

Well, I wouldn't know what else to do with my life. It's like, “What am I actually living for? And why do I have all these privileges and a good education and a supportive environment? If not to work towards making things better for everyone?” (Sebastian, 29)

To get to this point, however, it seems relevant to first understand the dimension of inequality and one's own implication in it, which, as Sina (28) shared, can be a painful process of (un‐)learning:

And then, when I took this seminar, these first seminars, and I also showed very strong defence mechanisms when it came to dealing with racism, I read Noah Sow's Deutschland Schwarz Weiß [Germany Black and White]. It took me a very long time to read this book, I needed a year and a half to read it. (Sina, 28)

The process of overcoming defensiveness and being open to learning about privilege therefore seemed painful, complicated, sometimes exhausting and time consuming. However, this process can also be appraised in a more positive manner: as an opportunity for moral growth (see Does et al., 2011, 2012).

I'm at a point right now where I'm trying to deal with things like that, with areas where I am privileged. For me, it's a learning process that never ends, and I'm always trying to learn more and see how I can be an ally to people who may be less privileged in this regard. How can I try as much as possible not to discriminate, try to show solidarity in a situation with people who have fewer privileges at this moment in time? (Charlie, 23)

The idea that dealing with privilege is a ‘learning process that never ends’ came up multiple times in the interviews. This indicates that some interviewees were able to manage identity threat with a flexible, growth‐oriented self‐identity. Learning more about privilege created an urge to facilitate this process in others as well. Carla (24), for example, stated that it is an important activism motivation for her to start a reflection process in others, regardless of its effectiveness for the overarching quest of dismantling privilege.

Learning about privilege, therefore, is an important process that provides meaning to privilege while activism provides opportunities to (un‐)learn harmful practices collectively. Triggering critical reflection in others and being accountable to others seemed to be an important motivating experience, which further links the quest for meaning to social justice activism. In sum, privilege self‐reflection, even though often considered to be painful and a life‐long process, is described as an important motivator for initial activism and for staying engaged. Given the previous themes, this process seems to require safe intrapersonal and interpersonal relations that can withstand one's critical curiosity.

#### Relativizing the role of privilege for activism

While most interviewees were generally open to the idea of reflecting on and learning about their own privilege, some questioned the relevance or even existence of social privileges. In the most extreme case, Rolf (74), an elderly White man, outright rejected being privileged, ‘I don't know how someone with privileges feels’. His rejection might, however, be attributed to his somewhat deviate representation of privilege (see above). Other interviewees wondered about the usefulness of the concept in the context of social change. Ute (42), for instance, was concerned about the ‘boundlessness’ of the topic and that getting held up in privilege discourse could prevent more pertinent discussions. In addition, Alex, Luisa and Oliver linked discourse around privilege to an emerging polarization of society. They were somewhat sceptical of the focus on privilege discourse within activist groups, because it could contribute to ‘political friction’ (Alex, 69) and ‘division’ (Luisa, 29), which supports the notion that even within social justice activism, social privilege can lead to defensiveness and conflict. Oliver (31) seemed to sympathize with this idea and shared his experience of a seminar in which privilege was discussed as ‘definitely not something like the starting point’ when it comes to social justice activism. At another point, he lamented that the discourse quickly ‘stops being about structural relations of oppression but about relative privileging’. However, it should be noted that when it comes to working together for social change, some friction and conflict on issues pertaining to inequality within the group may be preferable to maintaining harmony (see Dixon et al., 2012).

On another note of relativizing the importance of privilege for social justice activism, Marco (36) pointed out that class privileges mainly imply that the super‐rich have a special responsibility in relation to whom he felt to be able to contribute only little, ‘these three percent also have the power to change the status quo’. The power of these ‘three percent’, however, also rests on the complicity of those who have the most to lose in opposing them: a relatively privileged middle‐class. As such, this perspective risks alleviating a large and powerful group from the responsibility to work towards equality. While a wider discussion about the usefulness of the privilege discourse within activism groups is beyond the scope of this paper, we want to note that previous research found privilege awareness to be an important criterion when people of disadvantaged groups were asked what differentiates ‘friends’ from ‘allies’ (Brown, 2015).

## DISCUSSION

A growing body of research shows that how people from advantaged social groups react to confrontations with their privilege depends on the specific identity threat that privilege elicits (Eckerle et al., 2023; Knowles et al., 2014; Lowery et al., 2007; Phillips & Lowery, 2020) and the resources at their disposal to overcome identity protection strategies (Bergkamp et al., 2022). We approached this phenomenon using identity process theory as a tool, which expects that ‘individuals construct systems of meaning for making sense of their lives, experiences and identities’ (Jaspal, 2014, p. 5). Understanding how the meaning‐making of privilege relates to the lived experience of allyship and social justice activism can shed light on the threats and identity processes involved when reconciling with privilege. We therefore sought to explore the social representations of privilege among allies, the identity processes that these representations trigger, and how privilege relates to their activism motives.

The activists we interviewed agreed (with some exceptions) on general definitions of social privilege, in that they are the result of structural inequality regarding the distribution of resources, connected to arbitrary identity features, and that social privilege is normalized and therefore often invisible. However, activists had differing understandings of the origin and maintenance of social privilege. While some highlighted their own inadvertent role in maintaining the underlying systems of inequality by virtue of their social identities (a complex representation), others seemed to conceptualize privilege more as a unidirectional flow from systemic arrangements to social identities (a unidirectional representation). These two competing social representations of social privilege imply different forms of meaning‐making regarding activism. While the complex representation implies inevitability of one's complicity in an unjust system, the unidirectional representation implies that activism can liberate oneself from responsibility for the status quo. We therefore speculate that these two representations of social privilege have downstream consequences on the continued motivation to engage in ‘privilege checking’ and discourse around social privilege within the activist group.

We further identified three categories of identity threat connected to privilege, specifically concerning its threat to moral integrity, reflections about the inevitability of privilege (positionality threat), and its consequences for social relations (social threat). These threats seemed pertinent and relevant to the everyday political work of allies. In some ways, this challenges previous models of allyship motivation in relation to social privilege (Bergkamp et al., 2022; Knowles et al., 2014; Radke et al., 2020), as engaging in allyship itself does not seem to suffice to successfully manage identity threat. In fact, engaging in allyship sometimes even exacerbates and complicates privilege identity threat (such as in the case of worrying about reproducing narratives of White saviourism). Both positionality and social threats imply perceived threat on dimensions, which are usually associated with social disadvantages (i.e. not being able to escape one's social class and limited access to privileged spaces).

Finally, we identified multiple themes that indicate how privilege, even though it was perceived predominantly as identity threatening, can be integrated into the activist identity: Social privilege was considered an opportunity and a resource to become active, from which seemed to flow a (moral) responsibility to act and to learn (more) about how social privilege works. In terms of identity process theory, the assimilation–accommodation of privilege and the activist identity seems to depend less on allies' ability to reduce its threat to their identity, but in their ability to channel the threat into an empowering, moral and meaningful behaviour. However, there were also some accounts of relativizing privilege and questioning the use of privilege reflection in social justice activism, which highlights that the process is not essential for social justice behaviour and that activists can be motivated by many factors beyond privilege reflection (see Radke et al., 2020).

### Insights and theoretical integration

To a large degree these results are in line with the finding that the path to allyship is lined with identity threats, which require resources such as interpersonal support, as well as the idea that this process is non‐linear and recursive (Bergkamp et al., 2022). However, our results also suggest that the previous conceptualisation of identity threat as thresholds that need to be overcome is incomplete. Some allies are active despite explicitly realizing that they will not be able to overcome threat borne from their social privilege. They pointed out that dealing with privilege is a life‐long learning process, which indicates both the awareness of the fact that the topic will continue to be identity threatening and the willingness to extract meaning and motivation to act upon this realization. For them, it seems that the psychological reconceptualization of identity threat as *manageable through action and growth* plays a key role in the process of assimilation–accommodation.

Our findings also challenge some existing models which are mainly built on quantitative research (Knowles et al., 2014; Shuman et al., 2024; van Zomeren et al., 2018) and thus often assume a rather rigid causality from cognition to collective action (see Vestergren et al., 2026). First, we found that advantaged group members can engage in political solidarity while still being unaware or rejecting their social privileges, which speaks to the idea that there are many different motives behind allyship (Radke et al., 2020). In addition, there were some accounts of increased privilege reflection *through* activism and relevant life events, which means that privilege awareness can also be an outcome of allyship, highlighting the dynamic nature of the integration process. Second, the present research points to a coping strategy via critical education that previous social psychological research under‐emphasized. That is, activists shared their motivation to learn more about their own privilege and educate others about privilege to preserve a positive identity. This role of the *meaning* motive surfaced in multiple places, suggesting a feedback loop of critical education, moral convictions, and activism. Third, we also learned that meritocratic threat can lead to the motivation to dismantle inequality when this threat is re‐appraised as a moral responsibility to use one's social privilege for good. This stands in contrast to previous research which considered meritocratic threat an exclusively negative predictor of dismantling inequality (Knowles & Lowery, 2012; Phillips & Lowery, 2020). Fourth, and in contrast to influential cognition models of political solidarity (Hamann et al., 2024; van Zomeren et al., 2008), collective efficacy was sometimes explicitly discounted as consideration for activism motivation. Instead, morality concerns seemed to motivate action even in the absence of efficacy beliefs (see Acar et al., 2024).

Beyond these insights, our findings illuminate dynamic motives behind allyship. Influential allyship models (Radke et al., 2020) situate privilege awareness within an outgroup focused motivation. In extension, the present research suggests that privilege reflection can be motivated by both ingroup and moral motives, and that it can be triggered by critical experiences. Specifically, interviewees shared how feelings of guilt triggered by experiences with members of disadvantaged outgroups encouraged them to reflect on their own privilege (although sometimes guilt also seemed to lead to withdrawal from the outgroup). We therefore argue that allyship can then be understood as a strategy to negotiate critical experiences that highlighted social privilege when given appropriate resources to overcome identity protection strategies (Bergkamp et al., 2022; see also Landmann et al., 2023). In this sense, allyship may play an important role in the accommodation–assimilation process of the new social representation of ingroup privilege created by critical experiences.

The present research also provided insight on the relevance of the coherence motive in integrating social privilege and activist identity. When privilege reflection clashes with moral identity, this seemed to trigger a motive to preserve identity coherence (Jaspal & Cinnirella, 2010). In the research, where the coherence motive was first postulated, coherence was investigated within gay Muslims' need to reconcile their sexual identity (e.g. being gay) with conflicting religious beliefs (e.g. being Muslim). In our case, the issue is somewhat flipped on its head: Coherence was aimed at reconciling one's privilege with one's moral beliefs about social justice. Thus, we argue that the coherence motive is activated when two identity elements are in *moral* conflict. In this sense, identity coherence may boil down to the question ‘How can I live with myself in light of this new information?’ This perspective would link the coherence motive with research on moral improvement (Leach & Iyer, 2024), future thought (Oettingen, 2012) and, on a collective level, collective future thinking (Kashima et al., 2025), which suggests that mentally contrasting an ideal (moral) future self or collective with the present (morally flawed) self or collective can activate change motives.

### Implications and future research

Regarding interventions, our results suggest that it may be fruitful to link privilege reminders to egalitarian moral values, for example, by framing the privilege reminder as an opportunity for moral growth instead of a threatening moral obligation (see Does et al., 2012) or by highlighting privilege through evidence of outgroup discrimination (Uluğ & Tropp, 2021). Critical exposure of this kind may help circumvent identity threat and thus kneejerk reactions to inequality in defence of one's social identity (see Bergkamp et al., 2022). Future research could investigate these self‐ and collective improvement motives as quests for identity coherence to better understand allyship behaviour.

Our findings also connect to previous research that showed that the experience of life events which highlight inequality and de‐normalize the status quo can motivate political activism (Lim & DeSteno, 2016; Thomas et al., 2022) and critical privilege reflection (Uluğ & Tropp, 2021). Such *key experiences* (Landmann et al., 2023) can challenge social representations (e.g. about racial inequality) and thus activate assimilation–accommodation and evaluation processes (e.g. reconciling one's White identity with the new representation). Future research may test and expand this application of IPT to collective social change (see also de la Sablonnière & Usborne, 2014). Further, the present research demonstrates the importance of critical education, because it provides opportunities to disrupt the narrative of a just and equal status quo (see, e.g. Bonam et al., 2019; Goudarzi et al., 2022).

### Limitations

Our intention with this research was to explore the integration of social representations of privilege and the social justice activist identity. We believe that a qualitative approach was best suited to explore this question, especially as qualitative research on people's understanding of their social privilege and collective action is rare. The interviews have led to a rich and complex account that aligns with and expands previous research on allyship motivations (Phillips et al., 2024; Radke et al., 2020) and coping with privilege (Bergkamp et al., 2022; Knowles et al., 2014; Shuman et al., 2024). Still, there are some shortcomings of the present research.

It is important to highlight that the role of the privileged in the fight for social equality involves perils for social movements (Droogendyk et al., 2016; Spanierman & Smith, 2017) and that the presence of allies in such movements seems to have surprisingly little effect on its capacity to achieve change (Hartwich et al., 2023). We therefore emphasize that the present research does not speak to the question how allies in social movements should behave or whether their participation is valuable for social justice movements. In contrast, our research suggests that reflecting on own social privilege makes certain identity motives salient, which can be more or less helpful for the movement cause. More self‐centred motives, such as self‐efficacy, self‐esteem and meaning, for example, may become problematic, when they imply a need for power or approval by the disadvantaged and may thus take resources away from the cause (which some interviewees also recognized). It would be interesting to see whether greater importance of the coherence motive (i.e. reconciling privilege with social justice values) in relation to self‐centred motives (i.e. maintaining self‐esteem, self‐efficacy and meaning) has positive consequences for how allies behave within activist organizations (see also Phillips et al., 2024).

Furthermore, we did not differentiate between different dimensions of privilege. While White privilege was perhaps most salient because forced migration‐related activism was the most prevalent form of activism in the sample, other forms of privilege (e.g. class‐based) were relevant as well (and often several forms intersected). In fact, allies themselves did not differentiate much between different systems of oppression and privilege. While these systems do have many commonalities (Nixon, 2019), the historical and contextual circumstances need to be accounted for to provide a complete picture of inequality (e.g. colonialism in the context of White privilege).

## CONCLUSION

While previous research has mainly focussed on privilege awareness as a *predictor* of allyship, we focussed on the meaning‐making of social privilege among those who are already politically active, thus emphasizing the dynamic nature of privilege representations and activist identity. Specifically, we investigated, based on social representations (Moscovici, 1961) and identity process theory (Jaspal & Breakwell, 2014), how advantaged social justice activists represent their social privileges and the process by which this representation interacts with their activist identity. Through a thematic network approach (Attride‐Stirling, 2001), we identified three global themes which contribute to the experience of an advantaged social justice activist identity: *making sense of privilege*, *how privilege threatens identity* and *relating privilege to activism*. Together, these themes delineate how privilege is integrated into the activist identity, how its representation relates to activism motivations and how it is shaped by experiences made during activism. A key insight was the importance of (moral) responsibility borne from being privileged and associated identity threats. This responsibility seemed to relate to the coherence motive, which provided a link between the threat of profiting from undeserved social advantages in a system of inequality, and the motivation to dismantle this system. Further exploration of this process can foster theory building about the recursive relationship between privilege awareness and allyship. The present research can also contribute practical knowledge that can help activists in understanding the complications that are likely to emerge from the reproduction of systems of inequality within their movements.

## POSITIONALITY STATEMENT

The study was motivated by the desire to better understand how privilege relates to political activism. As White scholars employed at German universities holding the citizenship of the country we live in, we can be considered privileged in many respects. The people we have interviewed for this study are active in different human rights and political organizations. We are not members of these organizations, nor do we consider ourselves advantaged activists. We do identify with their social justice orientation, which we also pursue in our own social psychological research exploring intergroup conflict, migration experiences, collective victimization, privilege and the reappraisal of past and present injustices.

## AUTHOR CONTRIBUTIONS

**Frank Eckerle:** Conceptualization; data curation; formal analysis; investigation; methodology; visualization; writing – original draft; writing – review and editing. **Carmen S. Lienen:** Conceptualization; investigation; methodology; validation; writing – original draft; writing – review and editing. **J. Christopher Cohrs:** Supervision; writing – review and editing.

## Supporting information


Data S1:

## Data Availability

Anonymized interview transcripts can be made available upon request. Ethical considerations for data protection make public sharing inappropriate.

## Footnotes

^1^
Seven interviews were collected as part of a Master thesis. The student was offered but declined authorship.

^2^
Germany also uses an idiographic term for capitalism which translates to social market economy, *soziale Marktwirtschaft*, meaning a more benign version of capitalism with a stronger social security net.
