# Supplementary material for: ‘A learning process that never ends’: How advantaged social justice activists negotiate privilege and activism within their identity
Source: Br J Soc Psychol. 2026 Apr 2;65(2):e70077. doi: 10.1111/bjso.70077 (PMC13046892; doi:10.1111/bjso.70077)
Supplement: Supplementary file 1 — Data S1: [file BJSO-65-0-s001.docx]

# “A learning process that never ends”: How advantaged social justice activists negotiate privilege and activism within their identity.

*Supplementary material*

## Interview guide

### Part I Political activism

1. Can you tell me the story of how you became politically active?
2. What motivates you to become politically active?

#### Supplementary questions:

- Area: In what area are you politically active?
- Goals: What are the goals of the political organisation you are involved with?
- Target group: What is the target group you want to help?
- Motives back then: If you think back to the time when you decided to become politically active, what were your motives for doing so?
- Motives today: What drives you to continue being politically active?
- Effectiveness: Do you feel that your political involvement has made a difference?
- Target group connection: What connects you to the target group you want to help? Do you have personal relationships with people who belong to the target group? Do you see a relationship between your experiences and the experiences of the target group?
- Morals and values: To what extent do moral principles or spiritual values play a role (in relation to their political commitment)?
- Meaning: What meaning does political activism have for you?
  - Self-interest: To what extent do you yourself benefit from your political involvement?
  - Self-image: Does your involvement have an influence on how you see yourself?
- Belonging to the politically active group: What significance or importance does the group with which you are politically active have for you? To what extent do you feel that you belong to the organization in which you are active?
- Affiliation with other groups: How do other people in your close react to your political activism?

*Transition from Part I to Part II: “You have now told me a lot about your political commitment. I would now like to move on to another topic, namely privilege.”*

### Part II Privileges

1. Tell me, what do you associate with the term privilege?
2. Definition: What does privilege mean to you? What do you think it means to be privileged?
3. Origin: How do you think people get their privileges?
4. Imprint: How is the topic discussed in your environment?
5. Forms: What types of privilege do you know?
6. Evaluation: How do you evaluate privileges? What are the advantages/disadvantages of being privileged?
7. Significance for self: What significance do privileges have for your self-image or the way you see yourself as a person?
8. Significance for others: What meaning do you think privilege has for others?
9. Visibility: To what extent are privileges generally visible or invisible (or unconscious)? In what situations have you experienced the topic of “privilege” being discussed? Do you yourself engage in a confrontation or self-reflection with the topic?
10. Emotions: What kind of feelings does the topic of privilege trigger in you? How would you evaluate the topic from an emotional point of view?

*Transition from Part II to Part III: “Now that we have discussed the topic of privilege, I would like to bring together the two topics of ‘privilege’ and ‘political activism’ in the following question to you:”*

### Part III Privilege and political activism

1. To what extent do you think there is a connection between privilege and political activism?
2. What do you think of the idea of reducing privilege (through political activism)?

### Part IV Conclusion

1. What do you take away from the interview?
2. Have we forgotten anything you would like to mention?
3. Insights and (new) thoughts: What thoughts did the interview possibly trigger?
4. Emotions:
   1. During the interview: What feelings were present during the interview?
   2. Afterwards: How do you feel right now?

## Overview of political groups

#### Seebrücke

Seebrücke is a civil society movement that campaigns for a migration policy based on solidarity and human rights. The movement calls for a revision of German and European asylum and migration policies and organizes demonstrations and protests all over Germany.

<https://www.seebruecke.org/ueber-uns>

#### Seawatch

Seawatch is a civilian sea rescue organization. Its purpose is to rescue refugees who are in distress at sea while crossing the Mediterranean.

<https://sea-watch.org/ueber-uns/>

#### Amnesty International

Amnesty International is a global organization that campaigns for human rights. It is politically independent and its campaigns and actions are based on the Universal Declaration of Human Rights.

<https://www.amnesty.de/amnesty/wer-wir-sind>

#### Zugvögel

Zugvögel is a German association that sees itself as a political organization that addresses post-colonial power imbalances. The association engages in educational work on the topics of partnerships in the context of global power inequalities, global freedom of movement and colonial continuities.

<https://zugvoegel.org/selbstverstaendnis/>

#### Lesbian and Gay Association Germany

The Lesbian and Gay Association in Germany (LSVD) is a civil rights association and represents the interests and concerns of lesbian, gay, bisexual, trans*, intersex and other queer people (LSBTIQ*).

<https://www.lsvd.de/de/home>
